# Perceptions on acceptability of the 2016 WHO ANC model among the pregnant women in Phalombe District, Malawi – a qualitative study using Theoretical Framework of Acceptability

**DOI:** 10.1186/s12884-023-05497-6

**Published:** 2023-03-11

**Authors:** Prince Nyumwa, Agatha Kapatuka Bula, Alinane Linda Nyondo-Mipando

**Affiliations:** 1 Department of Health Systems and Policy, School of Global and Public Health, Kamuzu University of Health Sciences, P/Bag 360 Blantyre 3, Blantyre, Malawi; 2 Holy Family College of Nursing, P.O. Box 51224, Limbe, Malawi; 3 University of North Carolina (UNC) Project, P/Bag A-104, Lilongwe, Malawi; 4 Maternal and Fetal Health Group, Malawi Liverpool Wellcome Programme, P.O Box 30096, Blantyre, Malawi

**Keywords:** Perceptions, Acceptability, Antenatal care, 2016 WHO ANC model

## Abstract

**Background:**

The World Health Organization introduced a new model of care, ‘The 2016 WHO ANC Model’ to overcome challenges encountered during the implementation of the Focused Antenatal Care Approach. For any new intervention to achieve its objective, it must be widely accepted by both the deliverers and recipients. Malawi rolled out the model in 2019 without carrying out acceptability studies. The objective of this study was to explore the perceptions of pregnant women and health care workers on the acceptability of 2016 WHO’s ANC model in Phalombe District, Malawi using the Theoretical Framework of Acceptability.

**Methodology:**

We conducted a descriptive qualitative study between May and August 2021. The Theoretical Framework of Acceptability was used to guide the development of study objectives, data collection tools, and data analysis. We purposely conducted 21 in-depth interviews (IDIs) among pregnant women, postnatal mothers, a safe motherhood coordinator, and Antenatal care (ANC) clinic midwives, and two focus group discussions (FGDs) among Disease Control and Surveillance Assistants. All IDIs and FGDs were conducted in Chichewa, digitally recorded, and simultaneously transcribed and translated into English. Data was analysed manually using content analysis.

**Results:**

The model is acceptable among most pregnant women and they reckoned that it would help reduce maternal and neonatal deaths. Support from a husband, peers, and health care workers facilitated acceptability of the model while the increased number of ANC contacts which resulted in fatigue and increased transportation cost incurred by the women was a deterrent.

**Conclusion:**

This study has shown that most pregnant women have accepted the model despite facing numerous challenges. Therefore, there is a need to strengthen the enabling factors and address the bottlenecks in the implementation of the model. Furthermore, the model should be widely publicised so that both intervention deliverers and recipients of care implement the model as intended. This will in turn help to achieve the model’s aim of improving maternal and neonatal outcomes and creating a positive experience with health care among pregnant women and adolescent girls.

**Supplementary Information:**

The online version contains supplementary material available at 10.1186/s12884-023-05497-6.

## Introduction

Malawi, just like many other sub-Saharan African countries, has the highest maternal mortality ratio (MMR) and neonatal mortality rate (NMR) estimated at 439 maternal deaths per 100,000 live births and 27 neonatal deaths per 1,000 live births respectively [1]. The provision of antenatal care (ANC) services is a primary strategy that facilitates early detection and prevention of most complications and existing diseases in a pregnant woman which in turn help reduce MMR and NMR [2, 3]. To support the acquisition of full benefits from ANC services, the World Health Organization (WHO) released comprehensive guidelines that have a model, the “2016 WHO ANC Model” for the routine management of pregnant women and adolescent girls at ANC clinics [4]. The model recommends that pregnant women should attend a minimum of eight ANC contacts, an increase from the preceding guidelines that had four visits [4]. According to WHO [4], the new model recommends that the first contact takes place in the first trimester (up to 12 weeks of gestation), two contacts scheduled in the second trimester (at 20 and 26 weeks of gestation), and five contacts scheduled in the third trimester (at 30, 34, 36,38 and 40 weeks of gestation). WHO [4] contends that the increased number of contacts provides pregnant women with a positive pregnancy experience and further creates an opportunity for health workers to know the pregnant women better and detect any potential complications.

In response to the WHO recommendations, Malawi rolled out the model in July 2019 [Phalombe Safe Motherhood Coordinator, 2020 personal communication, May 11] without carrying out acceptability studies. However, WHO identified a strong relationship between the acceptability of a health intervention and service utilisation whereby acceptable services are more likely to be utilised, unlike unacceptable services which are less likely to be utilised as intended even if they are available and accessible [5]. Malawi’s environment for implementation of this policy is challenged by the high levels of MMR and NMR [1], high levels of poverty [6], and shortage of skilled medical personnel that results in a high provider-to-patient ratio [7], and a shortage of infrastructure [6]. The fragility of the environment necessitated the understanding of the acceptability of this new model and provide policymakers and healthcare workers with strategies for optimising the implementation of the model. There was limited information on the acceptability of the model among pregnant women in Malawi including the factors that influence pregnant women’s decision whether to utilise the ANC services or not. Given the gap in knowledge and the influence of the acceptability of a health intervention on service utilisation [5], this study explored the perceptions of pregnant women and health care workers on the acceptability of 2016 WHO ANC model in Phalombe District.

The study was guided by a Theoretical Framework of Acceptability (TFA), which is a multi-faceted construct that reflects the extent to which people delivering or receiving health care interventions consider it to be appropriate based on anticipated or experiential cognitive and emotional responses to the intervention [8]. The constructs of the TFA include Affective Attitude, Burden, Ethicality, Intervention Coherence, Opportunity cost, Perceived Effectiveness, and Self-efficacy [8]. This theoretical framework was used to assess acceptability prospectively, concurrently, and retrospectively [8]. To achieve a prospective analysis, we assessed pregnant women’s acceptance of the model and their intention to adhere to the prescribed ANC schedule at the initial ANC contact. Concurrent acceptability was assessed among pregnant women at their eighth contact to elicit factors that were helping them continue adhering to their schedule. To achieve a retrospective analysis, we included postnatal mothers and explored the factors that enabled them to complete or not complete the ANC schedule and how they accepted the model based on their experience with it. TFA also guided the development of the study objectives, the designing of focus group discussion (FGD) and in-depth interview (IDI) guides, and the analysis of the data.

## Methods

### Study design and setting

This was a descriptive qualitative study design that allowed for the solicitation of participants’ perceptions cognisant that acceptability is a subjective evaluation made by individuals who are experiencing or have experienced the intervention [8]. The study took place at Phalombe Health Centre in Phalombe District, located in the Southern Region of Malawi. The district was chosen because of its rural location, which poses a challenge to the attendance of ANC services [1]. According to the 2015/16 Malawi Demographic Health Survey (2015/16 MDHS) [1], rural districts in Malawi had an attendance of ANC services at 49% compared to 59% of their urban counterparts. The 2015/16 MDHS, further showed that Phalombe District had the highest NMR of 40 neonatal deaths per 1000 live births, which was above the national rate of 27 neonatal deaths per 1000 live births [1], and post-neonatal mortality rates at 28 deaths per 1,000 live births which were among the third highest districts in Malawi [1]. Phalombe District had a population of about 429,450 with 14 out of 15 health facilities offering ANC services. The district rolled out the 2016 WHO ANC Model in August 2019 in all the health facilities. At the time of data collection, that is from May to August 2021, Phalombe Health Centre was the district’s main public health facility as there was no functional district hospital. The district relied on Holy Family Mission Hospital for all in-patient care where admitted patients accessed care for free through Service Level Agreement with the Malawi Government.

The staff at Phalombe Health Centre consisted of medical officers, clinical officers, clinical technicians, medical assistants, nursing and midwifery officers, nurse midwife technicians, radiology technicians, dental technicians, environmental health officers, and disease control and surveillance assistants. The health facility offered a wide range of outpatient services such as antiretroviral therapy, adult outpatient care, mental health, orthopaedics, laboratory, radiology, pharmacy, ophthalmic and maternal and child health services. Three midwives who were permanently allocated at the facility’s ANC clinic mainly provided uncomplicated ANC services including ultrasound-scanning services.

The researchers chose Phalombe Health Centre because it was the main public referral facility in the area and presented a pool of clients for the researchers to draw from. The researchers believed that the participants drawn from Phalombe Health Centre were a representation of all the women in the district as they had similar socioeconomic and demographic health statuses.

### Sampling and sample size

We purposely selected and interviewed thirty-three (33) participants based on their age, pregnancy status, parity, expert knowledge and role in the provision of ANC services, and their willingness to participate in the study [9, 10] (see Table S1). The participants included; pregnant women (both at initial and 8^th^ contact), postnatal mothers (both completed and those that did not complete their schedule), and health care providers that included disease control and surveillance assistants (DCSA), ANC clinic midwives, and the safe motherhood coordinator (see Table S1).

Women were recruited during their scheduled ANC and postnatal visits. ANC clinic midwives and postnatal ward midwives assisted in the identification of pregnant women and postnatal mothers respectively and briefed them about the study. Those interested were referred to the Principal Investigator (PI) who explained the purpose of the study and obtained written informed consent. The maternity unit in charge facilitated the selection of ANC clinic midwives and a safe motherhood coordinator. Senior DCSA facilitated the selection of eligible DCSAs. Of all the participants approached, two postnatal mothers, particularly the multipara, refused to take part in the study citing time constraints and unwillingness as their reasons. Recruitment continued until there was data saturation [9].

### Data collection

Data were collected from May to August 2021 through face-to-face in-depth interviews (IDIs) and focus group discussions (FGDs). A total of 21 IDIs were conducted with pregnant women, postnatal mothers, safe motherhood coordinators, and ANC clinic midwives, and two FGDs were conducted with DCSAs (see Table S1). Data was collected using semi-structured interview guides that were informed by the Theoretical Framework of Acceptability. The guides were reviewed by ALNM and AKB who have experience in maternal health issues and qualitative research. The tools were pilot-tested at Migowi Health Centre in the same district and the findings helped to modify the data collection tools [11]. The PI conducted all the IDIs and FGDs and two research assistants, experienced in qualitative research, assisted with note-taking during FGDs. All IDIs for pregnant women and postnatal mothers and FGDs were conducted in Chichewa (the predominant local language), while interviews with midwives were conducted in English. All interviews were audio-recorded and lasted for approximately one hour and two hours respectively.

The research team ensured the quality of the data was credible, transferable, and reflexible [10, 12, 13]. The credibility of the data was achieved by summarising the key findings at the end of the interviews and discussions as a form of member checking [10]. The detailed description of the research methodology, setting, study findings, and verbatim quotes from individual interviews maximised the applicability of the study to other similar contexts [10, 12]. ALNM and AKB randomly read selected transcripts to identify major categories, so that readers may have a clear picture of the findings, and provided continuous feedback to the data collection team. Reflexivity of this study was achieved through the use of an audit trail [13], where an interpretation matrix was developed, which contained major themes and subthemes supported by quotes, which can be verified if other researchers decide so.

### Data analysis

The audio recordings were transcribed verbatim directly into English. Each transcription had a unique identification code that was assigned to the participant during the interview. A codebook was developed following a review of three transcripts by the research team (See Table S4). Data was analysed manually where all transcripts were read while applying the codebook and keywords according to the TFA were captured in the margins. Manual data analysis ensued following a directed content analysis technique [14] where data was deductively coded using the seven constructs of the Theoretical Framework of Acceptability (TFA) [8]. Once all transcripts were coded, the researchers examined all the data within a particular code. Data that was under the same code but appeared to contain different ideas was split into subcategories. Whereas data that appeared in different codes but had similar ideas was combined into one subcategory [14, 15]. Finally, the research team developed a matrix of themes, subthemes, and direct quotes from the study participants to the matrix.

### Ethical consideration

The College of Medicine Research and Ethics Committee (COMREC) (P.01/21/3240) provided the ethical approval for the study while the Director of Health and Social Services (DHSS) for Phalombe District Council granted the institutional support for the study to be conducted. All study participants signed or thumb printed a copy of the informed consent form before participating in the study. We ensured that all methods were carried out following relevant guidelines and regulations and that we did not deviate from the approved protocol.

### Study findings

#### Demographic characteristics of study participants

The demographic characteristics of our study participants showed that most of the women were young mothers aged between 18 and 24 years and started their ANC clinic in their first trimester (see Table S2). The table also shows that there were fewer postnatal mothers as well as the women that attended eight or more contacts as they were not readily available. The table further shows that out of the 18 women that participated in the study, seven were aware of the existence of the new model.

#### Characteristics of health care providers

The age range for health care providers was between 25 and 49 years with the majority falling between 41 and 49 years of age (Table S3). Unlike the DCSAs, most of the midwives who participated in this study were early career professionals, with less than two years of work experience. All 15 health care providers were aware of the new model of care, but only two had attended formal training on the model.

#### Perceptions on the acceptability of the 2016 WHO ANC Model

The concept of acceptability of the 2016 WHO ANC Model among pregnant women has been presented under seven constructs of the Theoretical Framework of Acceptability, which are Affective Attitude, Burden, Ethicality, Intervention Coherence, Opportunity Costs, Perceived Effectiveness, and Self-efficacy [8].

### Affective attitude

Affective attitude describes how an individual feels about an intervention [8]. Upon asking participants how they felt about the 2016 WHO ANC model, the majority of them stated that the number of contacts was adequate for proper monitoring of maternal and foetal wellbeing and early detection and management of problems. A pregnant woman at initial contact narrated:

“I am very pleased that we will be seen more frequently. Midwives will have more chances to monitor our wellbeing and that of our unborn babies, identify some problems, and manage them accordingly” (IDI_pregnant woman, 1^st^ contact).

The pregnant women’s positive feelings about the new model were influenced by the good attitudes displayed by the health care providers.

“Health care workers tried their best not to insult us. Had they been rude to us, we could have lost interest in coming back to this clinic and miss our next date of appointment. But the midwives were friendly.” (IDI_pregnant woman, 8^th^ contact).

Despite the majority of the women touting the model as a good one, few pregnant women, both primigravida, and multigravida did not like the increased number of contacts due to the challenges encountered while accessing services. A primigravida at her 8^th^ contact complained:

“Although the model entails good services, its schedule is not easy to complete. The previous model was good because of the reduced number of visits considering the distance the women have to endure to access the services. (IDI_pregnant woman, 8^th^ contact).

### Burden

Burden refers to the perceived amount of effort to participate in the intervention [8]. The women and health care providers highlighted long distances, increased transport costs, and fatigue as the major barriers associated with attending such an increased number of contacts. As a result, most of the pregnant women had problems adhering to and completing their ANC clinic schedule. A health worker narrated:

“The women that stay far away from the clinic have not accepted the model because for them to attend the monthly appointments means more cost to them. So, some women skip their appointments to avert the long distance and the increased transport cost” (IDI_ male DCSA).

A pregnant woman amplified the notion of fatigue that was associated with attending more contacts:

“When they began scheduling me frequently, I felt like I was being overburdened. Considering the long distance, I felt like I should not come particularly when I reached the 9th month when the appointment dates were so close to each other. I felt like I needed to rest” (IDI_ pregnant woman, 8^th^ contact).

### Ethicality

Ethicality refers to the extent to which an intervention has a good fit with an individual’s value system [8]. The majority of pregnant women and postnatal mothers explained that the care they received met their expectations and that they accepted the new model. The women affirmed this as they had their problems identified and managed. A postnatal mother who completed eight contacts and had lost a baby in the preceding pregnancy narrated:

“I feel pleased and have accepted this new model. All the problems I experienced during pregnancy were identified and managed during the encounter with the health care providers. The care I received helped me deliver a live baby without problems.” (IDI_ postnatal mother, completed 8 contacts).

In agreement, midwives felt the routine provision of the USS service would increase pregnant women’s attendance at ANC services.

“Provision of routine scanning services has the potential to improve acceptability because women like to be scanned to check the condition of the foetus inside the womb but also to check other problems that might be there other than those of the foetus. The women who have been scanned will develop high expectations and have a happy experience. In addition, these pregnant women who had pregnancy-related problems identified through abdominal USS and were managed promptly would motivate other reluctant women with similar problems to come and attend ANC clinics with the hope that they too would be scanned for their problems and be timely managed.” (IDI_midwife).

### Intervention coherence

Intervention coherence encompasses participants' understanding of an intervention and how it works [8]. The study participants expressed variations in their awareness of the existence of the new model. While few women reported having been informed about the existence of the new model, the majority denied their awareness of the model. A pregnant woman who completed her new schedule without knowing the existence of it narrated as follows:

“You mean there is a new model? I am not aware of this. What exactly has changed? Or maybe HIV testing because we are now being tested twice, or that we are attending more visits?” (IDI_ pregnant woman, 8^th^ contact).

Healthcare providers mentioned a lack of adequate knowledge of the model as most of them did not attend formal training and that there were no guidelines to guide their practice. Staff that were trained on the new model were rotated to other departments leaving out one midwife to train on the job the incoming new staff.

“I feel like I have little knowledge about this model because we were just told briefly about the services we have to offer to pregnant women. In addition, there are no guidelines here to guide our practice. So it is difficult for us to explain to the women for them to understand this model clearly.” (IDI_midwife).

### Opportunity cost

Opportunity cost refers to the extent to which benefits, profits, or values must be given up to engage in an intervention. [8]. In assessing opportunity costs, participants were asked to explain the competing interests that hinder pregnant women from accessing ANC services. The participants reported that being engaged in socio-economic activities, seeking traditional medicine, and fear of losing foetuses to witches were some of the factors that hinder pregnant women from starting ANC clinics early or completing ANC schedules. Furthermore, other women who develop minor disorders of pregnancy rely on traditional medicine to stabilise their pregnancy and delay initiating care once they get better.

“Some pregnant women who complain of abdominal discomfort seek relief from traditional healers or birth attendants where they are given concoctions to stabilise the pregnancy. Once they are cured, they do not see the importance of starting an ANC clinic or start the clinic very late while their pregnancies have advanced thereby minimising their chances of completing the new schedule.” (IDI_pregnant woman, 8^th^ contact).

### Perceived effectiveness

Perceived effectiveness refers to the extent to which the intervention is perceived as likely to achieve its purpose [8]. Most of the participants felt that the model has the potential to reduce maternal and neonatal mortality rates.

“I think the new model has a great impact in reducing maternal and neonatal mortality rates. This is because in every visit, we assess the woman and it’s very easy for us to note if something is wrong with the woman hence correcting any problems that may arise. In addition, the USS assists in the identification of some of the problems which we could have missed if using the old model. Furthermore, we provide these women with more doses of Fansidar SP hence protecting them from malaria.” (IDI_ Safe motherhood coordinator).

### Self-efficacy

This refers to participants’ confidence that they can perform the behaviour required to participate in the intervention [8]. The study found that some women completed their schedules. These women cited, apart from the positive attitudes of the health care providers, the presence of social support from their husbands and peers who adhered to and completed their ANC schedule as some of the factors that enabled them to complete the schedule. On husbands’ support, the women cited that their husbands wanted improved health status, good pregnancy outcomes, and pregnancy confirmation as some of the reasons for the husbands’ involvement in ANC services.

“Although I stay a bit far away from this health facility, my husband would provide transport for me to attend the clinic. He also reminded me about the appointment dates. My husband would not allow me to miss any clinic appointment date. He wanted to know the progress of our baby” (IDI_pregnant woman, 8^th^ contact).

On the other hand, women who failed to complete the schedule mentioned a lack of awareness of the model as the main reason for their failure.

“I was not aware that women were supposed to come to the clinic eight times so I just felt the three visits were enough. Now that I am aware of the new arrangement, I am ready to comply in the next pregnancy” (IDI_postnatal mother, attempted 3 contacts).

## Discussion

The main findings of the study are that the model is acceptable amongst most pregnant women and that it would contribute to the reduction of maternal and neonatal deaths. Most women were self-confident to complete the schedule. The major enabling factors to the acceptability of the model was the presence of support from husbands, peers, and healthcare providers. The increase in the number of ANC contacts is a major challenge because it resulted in fatigue and increased transportation costs incurred by the women.

The positive attitude expressed by the women towards the 2016 WHO ANC model, builds on findings from a Cochrane systematic review conducted in 2015 which showed that women in both low and high-income settings were less satisfied with the FANC’s reduced visit schedule perceiving the gap between visits as too long [16]. The women wanted adequate time to be assessed for complications, and interact with and learn from health care providers about their well-being and that of their child [4]. Additionally, a positive attitude from and the reported positive relationship that existed between the women and the healthcare providers in this study, corroborates findings from a systematic review carried out in 2015 [17]. In the review, a good staff attitude motivated pregnant women to use ANC services, it created higher self-esteem amongst the women using ANC services, and informed a woman’s decision to return to a facility on their next appointment date [17].

The satisfaction expressed by the women that were offered pelvic USS supports other previous studies that the provision of USS among pregnant women could create a positive pregnancy experience because it offered an opportunity to learn about the wellbeing of the foetus, early detection of complications or abnormalities, and prevention of procedures such as induction of labour later in pregnancy [4, 18, 19]. In another study carried out in Uganda, it was found out that women were motivated to attend ANC clinics when offered concrete incentives of seeing their baby through pelvic USS [20]. This highlights the need to accelerate the provision and advertisement of USS services so that women are aware of the service [20]. To ensure that USS is readily available there is a need to scale the training of midwives on basic obstetric ultrasound [21].

The negative effects of long distances to the health care facilities compounded by the lack of transportation to the clinic, in the present study, have also been reported in previous studies [22–24]. The previous studies found that an increase in distance to the nearest health care facilities results in fewer antenatal visits as pregnant women tend to reduce the number of antenatal contacts to reduce travel costs and fatigue [22–24]. Furthermore, the perception among some pregnant women that the increased number of contacts was a burden supports an earlier study carried out in Bhutan where it was reported that women who perceived the number of visits as excessive reduced the frequency of attendance at the clinic [25]. This calls for more efforts to increase women’s understanding of the rationale behind the increase in the number of contacts including the closeness of contact points in the third trimester [4]. Additionally, there is a need to conduct mobile clinics in hard-to-reach areas so that pregnant women access ANC services within their communities [26].

The adherence and completion of ANC clinic schedules among some pregnant women in the present study, despite being unaware of the schedule, complements a mixed study conducted in Bhutan which found that women who attach value to ANC services strive to adhere to their schedule [25]. On the other hand, WHO envisaged inadequate knowledge of the new model among the pregnant women as the most likely challenge to be faced during the implementation of the model [4]. This finding is also in tandem with an Ethiopian study which found that healthcare providers did not follow the guidelines, among others, due to lack of training, lack of mentorship program, and unavailability of ANC guidelines [27]. This, therefore, suggests the need for wide pieces of training on and dissemination of the model so that it is accurately implemented and the services are adequately utilised. [28–30].

Our findings that the study participants were optimistic that the 2016 WHO ANC model would help reduce maternal and neonatal mortality rates, support earlier findings by WHO which stated that frequent contact between pregnant women and health care providers would reduce stillbirths by up to eight per 1000 live births [4]. This is because the frequent contacts allow for early detection and management of pregnancy-related complications thereby reducing maternal and neonatal deaths [31, 32]. The study participants’ optimism about the effectiveness of the model also stems from the incorporation of the routine provision of pelvic USS to pregnant women which would aid in the detection of problems. This finding is consistent with the results from a cohort study carried out in Tanzania where a hand-held ultrasound scan (vScan) increased the detection of problems and compliance to referrals as the women had concrete evidence for their referral [33].

The factors that increased the high self-confidence among some pregnant women, in the present study, to adhere to and complete the new ANC schedule resonates with the findings of a study conducted in Iran which found that the presence of social support, especially from husbands, improved pregnant women’s ability to take care of themselves including attending ANC clinics [34]. The influence of peers in the present study supports the findings of a study conducted in Bhutan where pregnant women were influenced to initiate clinics and attend subsequent ANC contacts by their peers [25]. Similarly, Albert Bandura theorised that seeing others performing similar tasks as well as having verbal or social persuasion improves one’s self-efficacy [35]. Therefore, empowering women who completed their ANC schedule as peer educators has potential in increasing self-efficacy among other pregnant women [36].

Lastly, the challenges in finding an adequate number of women who completed eight or more contacts supports the current literature which indicates that few women, particularly in rural areas complete eight or more contacts [28, 37]. Cognizant of this, the authors probed more during the in-depth interviews to glean much information from these women as they could make up for the reduced numbers of potential participants.

## Strengths and limitations of the study

The use of the Theoretical Framework of Acceptability strengthened this study as it provided the lenses through which the researcher viewed the perceptions of the recipients of care (pregnant women) and the providers (health care providers) about the acceptability of the 2016 WHO ANC model. The researchers highlight a number of study limitations. One of them is that postnatal mothers were sampled less than pregnant women and that the women that completed their schedule were sampled less than those that did not. This was because these women were not adequately available or had refused to participate in the study citing time constraints. Therefore, future research should focus on the national prevalence of eight contacts or more to determine the number of women completing the new schedule. Another study limitation was that we used one facility as a study site such that the findings cannot be generalised to the entire district. However, the findings of this study can help local, national, and international policy makers to develop strategies that would ease the burden of accessing ANC services. Furthermore, the researchers did not include the women who defaulted from care. Therefore, future research should focus on barriers to adherence to the eight-contact schedule with a focus on women that defaulted from care. Finally, the authors acknowledge the limited size of the sample which limits its generalizability. Therefore, future research should aim at quantitative approaches with a larger sample size to assess the agreement with our findings.

## Conclusion

This study has drawn a mixed view of the acceptability of the 2016 WHO ANC model among pregnant women. While most of the recipients of ANC services accepted the model, viewing it as having the potential to provide them with the opportunity to regularly interact with health care providers, be examined for any potential problems, and be able to learn about health care from their providers, few mothers did not like the model’s increased schedule due to the burden associated with its completion. Therefore, addressing the bottlenecks of the implementation of the model will ease the burden of accessing ANC services. This will in turn help to achieve the model’s aim of improving maternal and neonatal outcomes and creating a positive experience with health care among pregnant women and adolescent girls.

## Supplementary Information

**Additional file 1:** 

**Additional file 2:** 

**Additional file 3:** 

**Additional file 4:** 

## Data Availability

The datasets used or analysed during the current study, including interview and study guides, are available from the corresponding author on a reasonable request.

## Abbreviations

ANC: Antenatal Care
COMREC: College of Medicine Research and Ethics Committee
DCSA: Disease Control and Surveillance Assistants
DHSS: Director of Health and Social Services
FGD: Focus Group Discussion
IDI: In-depth Interview
MDHS: Malawi Demographic Health Survey
MMR: Maternal Mortality Ratio
NMR: Neonatal Mortality Rate
PI: Principal Investigator
TFA: Theoretical Framework of Acceptability
USS: Ultrasound Scan
WHO: World Health Organization
