# Supplementary material for: Perceptions on acceptability of the 2016 WHO ANC model among the pregnant women in Phalombe District, Malawi – a qualitative study using Theoretical Framework of Acceptability
Source: BMC Pregnancy Childbirth. 2023 Mar 11;23:166. doi: 10.1186/s12884-023-05497-6 (PMC10007797; doi:10.1186/s12884-023-05497-6)
Supplement: Supplementary file 1 — Additional file 1: [file 12884_2023_5497_MOESM1_ESM.docx]

**Table S1 Summary of Methods**

| **Sample** | **Method** | **Purpose** | **Sample Characteristics** | **Sample size** |
| --- | --- | --- | --- | --- |
| Pregnant and postnatal mothers-multigravidae/multipara | In-depth Interviews | To provide their experiences with the new model at various contact points and to make a comparison with FANC policy. To provide factors that influence their continual or discontinuation of receiving ANC services | More than one time pregnant women at their initial contact and 8^th^ contact. Also includes more than one time mothers who accessed care at the facility (both completed and those that did not complete their schedule | 10 |
| Pregnant and postnatal mothers- primigravidae/primipara | In-depth Interviews | To provide their new experiences with the pregnant state and the new model at various contact points. To provide factors that influence their continual or discontinuation of receiving ANC services | First time pregnant women at either initial or 8^th^ contact point. They also included first time mothers who completed their ANC schedule and those that did not complete. | 8 |
| Safe motherhood coordinator | In-depth Interview | To provide key information on service provision, health systems facilitators, and challenges to the acceptability of the 2016 WHO ANC model (both clinic-based and community-based ANC) | This is a professional midwife overseeing delivery of maternal health services including ANC services within the district | 1 |
| Midwives | In-depth Interviews | To provide key information on service provision, health systems facilitators, and challenges to the acceptability of the 2016 WHO ANC model (clinic-based ANC) | Professional providers providing ANC services at the facility’s ANC clinic | 2 |
| DCSAs | Focus Group Discussions | To provide key information on service provision, health systems facilitators, and challenges to the acceptability of the 2016 WHO ANC model in the community (community based ANC) | Trained in community based maternal and newborn care. Providers of ANC related services at both the facility and community level | 12 (Two FGDs with six members in each group) |
