# Supplementary material for: Perceptions on acceptability of the 2016 WHO ANC model among the pregnant women in Phalombe District, Malawi – a qualitative study using Theoretical Framework of Acceptability
Source: BMC Pregnancy Childbirth. 2023 Mar 11;23:166. doi: 10.1186/s12884-023-05497-6 (PMC10007797; doi:10.1186/s12884-023-05497-6)
Supplement: Supplementary file 2 — Additional file 2: [file 12884_2023_5497_MOESM2_ESM.docx]

**Table S2: Showing Demographic Characteristics of pregnant women and postnatal mothers**

| **Participants (N=18)** | **Number (N)** | **% of total Participants** |
| --- | --- | --- |
|  |  |  |
|  |  |  |
| **Age** | | |
| 18-24 years | 12 | 67 |
| 25-30 years | 2 | 11 |
| 31-40 years | 4 | 22 |
| **Type of the women** | | |
| Pregnant women | 11 | 61 |
| Postnatal mothers | 7 | 39 |
| **Parity** | | |
| Para 0 | 6 | 33 |
| Para 1 | 6 | 33 |
| Para 2 or more | 6 | 33 |
| **Education Level** | | |
| No-formal | 1 | 6 |
| Primary | 11 | 61 |
| Secondary | 6 | 33 |
| College | 0 | 0 |
| **Occupation** | | |
| Business | 1 | 6 |
| House wife | 3 | 17 |
| Farmer | 14 | 78 |
| Employed | 0 | 0 |
| **Marital Status** | | |
| Married | 17 | 94 |
| Single | 1 | 6 |
| Separated | 0 | 0 |
| **Time of Initiating Antenatal Care** | | |
| First trimester | 12 | 67 |
| Second trimester | 6 | 33 |
| Third trimester | 0 | 0 |
| **Attempt of eight contacts** | | |
| Pregnant women | 4 | 36 |
| Postnatal mothers | 2 | 29 |
