# Supplementary material for: Perceptions on acceptability of the 2016 WHO ANC model among the pregnant women in Phalombe District, Malawi – a qualitative study using Theoretical Framework of Acceptability
Source: BMC Pregnancy Childbirth. 2023 Mar 11;23:166. doi: 10.1186/s12884-023-05497-6 (PMC10007797; doi:10.1186/s12884-023-05497-6)
Supplement: Supplementary file 3 — Additional file 3: [file 12884_2023_5497_MOESM3_ESM.docx]

**Table S3: Showing Demographic Characteristics of Health Care Providers**

| **Participants (N=15** |  |  |
| --- | --- | --- |
|  | **N** |  |
|  |  | **% of total** |
| **Age** | | |
| 25-30 years | 5 | 33 |
| 31-40 years | 4 | 27 |
| 41-49 years | 6 | 40 |
| **Gender** | | |
| Females | 11 | 73 |
| Males | 4 | 27 |
| **Education** | | |
| Secondary | 12 | 80 |
| College | 3 | 20 |
| **Occupation** | | |
| Safe Motherhood Coordinator (Nursing and Midwifery Officer) | 1 | 7 |
| Nurse and Midwife Technician | 2 | 13 |
| Disease Control and Surveillance Assistants | 12 | 80 |
| **ANC Experience** | | |
| 0-10 years | 6 | 40 |
| 11-20 years | 8 | 53 |
| 21-30 years | 1 | 7 |
