# Supplementary material for: Perceptions on acceptability of the 2016 WHO ANC model among the pregnant women in Phalombe District, Malawi – a qualitative study using Theoretical Framework of Acceptability
Source: BMC Pregnancy Childbirth. 2023 Mar 11;23:166. doi: 10.1186/s12884-023-05497-6 (PMC10007797; doi:10.1186/s12884-023-05497-6)
Supplement: Supplementary file 4 — Additional file 4: [file 12884_2023_5497_MOESM4_ESM.docx]

**Tab1e S4: Code book for perceptions on acceptability of the 2016 WHO ANC model using the vTheoretical Framework of Acceptability [8]**

| **Code/TFA Constructs** | **Description** | **Illustrative statements** |
| --- | --- | --- |
| Affective Attitude | Affective attitude describes how an individual feel about an intervention. used to discuss participants’ feelings about the new model of care | “I am very pleased with the model that we will be seen more frequently. Midwives will have more chances to examine us…” “When they began scheduling my contacts, I felt like I were being overburden…..I felt like I needed to rest.” “I was very delighted when I was scanned …..” |
| Burden | Burden refers to the perceived amount of effort to participate in the intervention. Used to describe what participants think pregnant women endure when the access care. | “When women think of the long distance they have to travel and the money they have to spend on transport they just think of postponing their visit to a later date……” |
| Ethicality | Ethicality refers to the extent to which an intervention has a good fit with an individual’s value system. Used for all discussion on how participants feel the model met their expectations | “I have accepted the model (she smiles)…..because most of my problems I had were solved. Had it been that it was during the previous model, where I was expected to attend only 4 visits, it meant these problems could have gone unnoticed.” “ |
| Intervention Coherence | Refers to the participants’ understanding of an intervention and how it works. Used for discussion on participants’ awareness of the model. | “I have known today that there is a new model, when I was initiating care, the health care providers had informed me.” |
| Opportunity Cost | Opportunity cost refers to the extent to which benefits, profits or values must be given up to engage in intervention. Used to discuss all what participants suggested that pregnant women had to give up as they access care. | “Some women fear that the witches would take away the foetus if it is exposed to the public early hence waiting for the foetus to grow and pregnancy stabilises probably at four months after the foetus has started foetal kicks.” |
| Perceived effectiveness | Perceived Effectiveness refers to the extent to which the intervention is perceived as likely to achieve its purpose. Used to discuss what participants think were the benefits of adhering to and completing the schedule, in addition to the discussion on the ability of the model to reduce NMR and MMR | “I think the new model has a great impact in reducing the number of maternal and neonatal mortality rates. This is because in every visit we usually assess the women and it’s very easy for us to note if something is wrong with this woman hence correcting any problems that may arise.” |
| Self-Efficacy | This refers to p participants’ confidence that they can perform the behaviour required to participate in the intervention. Used to discuss the participants’ abilities to complete or not complete the schedule. | “I am ready to complete the schedule, considering the way I have been welcomed today and the way the midwives have explained to me. I am ready to complete my schedule.” |
